# Squamous Odontogenic Tumor with Unusual Localization and Appearance: A Rare Case Report

**DOI:** 10.1155/2013/407967

**Published:** 2013-03-27

**Authors:** Sucheta Bansal, Sanjeev Kumar Joshi

**Affiliations:** ^1^Department of Oral and Maxillofacial Pathology, Himachal Institute of Dental Sciences, Paonta Sahib, Himachal Pardesh 173025, India; ^2^Department of Prosthodontics including Crown, Bridge, and Implantology, Himachal Institute of Dental Sciences, Paonta Sahib, Himachal Pardesh 173025, India

## Abstract

Squamous odontogenic tumor is a rare benign neoplasm and may affect multiple sites in the mouth. SOT was first described by Pullon et al. (1975). Since then, there have been less than 50 reported cases. The tumor is often asymptomatic, although it can present with symptoms of pain and tooth mobility. We report a case of SOT occurring in a 26-year-old female in the anterior mandible with unusual localization and appearance.

## 1. Introduction

Squamous odontogenic tumor (SOT) is a rare benign neoplasm first described in 1975 by Pullon et al. and now classified as an independent entity. It is thought to arise from a neoplastic change of epithelial rests of Malassez [1–3]. SOT is defined as a benign but locally infiltrative neoplasm which consists of islands of squamous epithelium in a fibrous stroma. Cystic degeneration or calcification may occasionally be observed in the epithelial islands [4]. 

SOT occurs mainly in the third decade of life and is approximately equally distributed between the maxilla and the mandible. In some cases, it may affect multiple sites in the mouth [5, 6]. Leider et al. [7] reported three cases of SOTs in siblings, which suggests a possible familial pattern in the occurrence of this lesion. Radiographically, SOT consists of a well-defined triangular-shaped radiolucent lesion adjacent to the roots of teeth [2]. The lesion is usually central, but sometimes it may be peripheral [8, 9].

Histologically, the tumor is characterized by multiple islands of squamous epithelium surrounded by a mature connective tissue stroma. The epithelial islands occasionally show foci of central cystic degeneration and calcifications [10]. The usual treatment has been simple enucleation, and recurrence has been rare [11]. 

Squamous odontogenic tumor is a rare tumor with nearly 50 reported cases till date [4]. The purpose of this paper was to report a clinical case of a patient with SOT with unusual location and the paucity of reported cases.

## 2. Case Report

**Figure 1:**
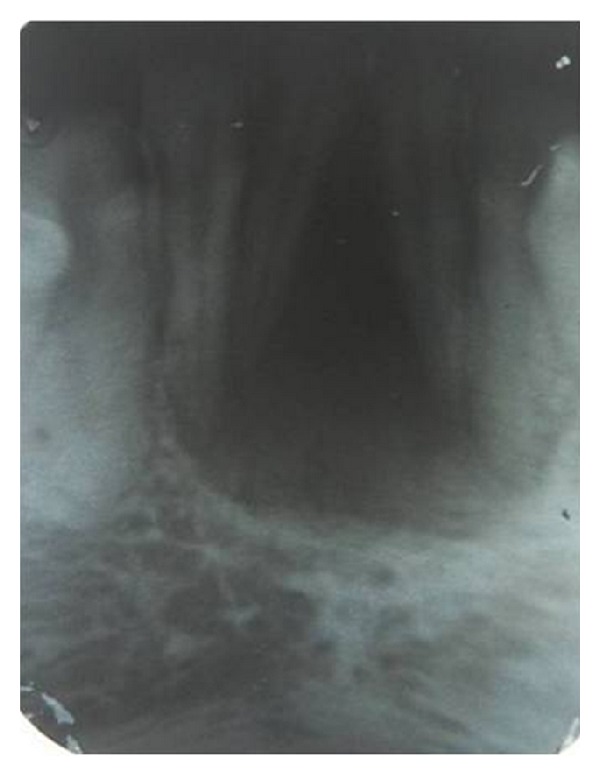
Intraoral periapical radiograph reveals triangular radiolucent area involving roots of mandibular central incisors.

A 26-year-old female patient reported to our dental hospital with a complaint of swelling in the anterior region of mandible for the past 11 months. Onset occurred 11 months ago and it was asymptomatic. On clinical examination, there was a firm anterior swelling in the central incisor region of mandible. The mandibular central incisors showed grade1 mobility. Radiograph showed radiolucency with severe alveolar bone loss localized between the diverging roots of the mandibular central incisors (Figure 1). 

**Figure 2:**
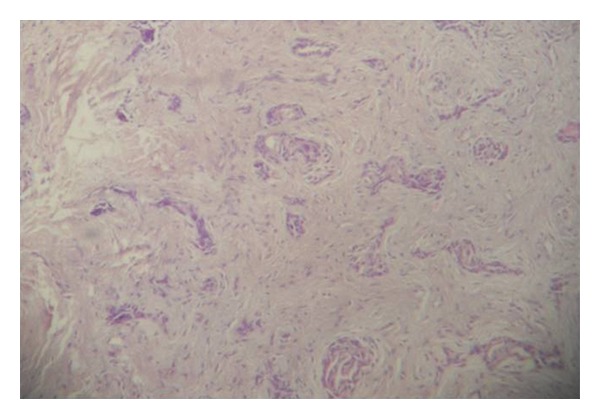
Histopathological picture showing odontogenic epithelial islands in mature fibrous stroma (4x magnification, H&E staining).

**Figure 3:**
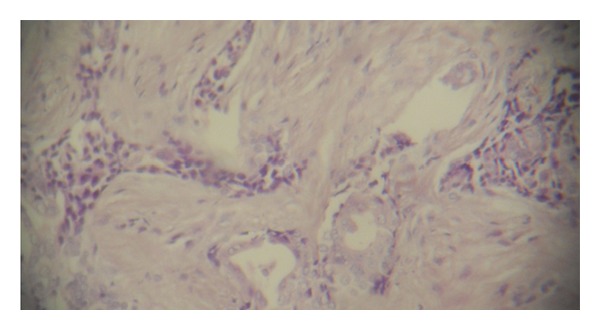
Histopathological picture showing odontogenic epithelial islands in mature fibrous stroma (40x magnification, H&E staining).

**Figure 4:**
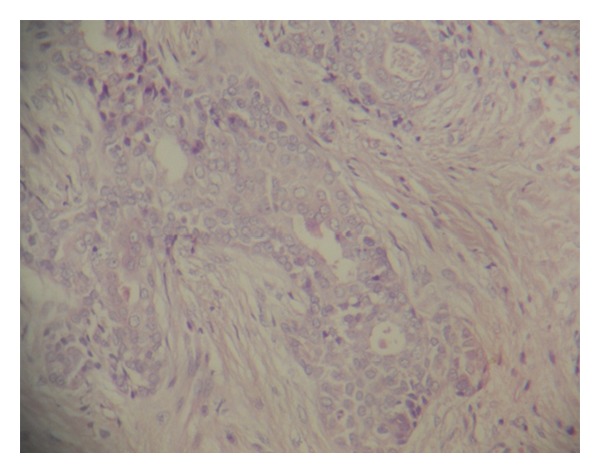
Histopathological picture showing microcysts formation within the epithelial islands.

The vitality test showed vital central and lateral incisors. Based on the clinical and radiographic findings, the initial diagnosis of periodontosis was made. The lesion was completely excised under local anesthesia and thorough curettage was done. The tissue was subjected for histologic examination. Histological examination revealed scattered islands of benign stratified squamous epithelial in dense fibrous connective stroma and the diagnosis of SOT was confirmed (Figures 2, 3, and 4). The patient reported back to the department first after 6 months then after 1 year and no evidence of recurrence was found. 

## 3. Discussion

Squamous odontogenic tumor is a rare lesion with few cases reported to date in the literature. Pullon et al. [1] were the first to describe it. They reported six cases and established diagnostic criteria and surgical approaches that are still followed today. Since that time, 45 cases of SOT have been reported [12]. Squamous odontogenic tumors have been found in patients whose ages ranged from 8 to 74 years (average age 38). The gender ratio among 44 cases is 1 : 1.8 (F : M) showing slightly more predilection among males.

Out of the 45 cases, only few cases have been located in anterior region of mandible especially in young females. In maxilla, SOT occurred most commonly in canine-first premolar area [13]. SOTs occurring in maxilla were found to be more aggressive than those in mandible [4, 8]. This was mainly due to the anatomy, porosity, and medullary nature of bone [9]. Hence, the most common location for development of an SOT in the maxilla is anterior region and posterior in case of mandible with almost equal propensity to occur in both the jaws [13]. Our case is unique in that it is localized between the roots of central incisors in the anterior segment of the mandible which is a rare location of occurrence. 

Clinically, SOTs represent as a slow growing lesion which leads to an increase in the volume of the maxillae or mandible, tooth mobility, ulceration of the soft tissue, painful symptoms, and tooth displacement. However, lesion may be asymptomatic and detected in routine intraoral radiographs [14]. Very few cases have been reported in the literature, and most of these have been located within the bone, although a few peripheral cases have also been discussed [2]. Radiograph of common central variant of SOT shows a well-defined unilocular, triangular radiolucency between the roots of adjacent teeth [9].

In our case, there was a firm anterior swelling in the central incisor region of mandible with grade 1 mobility and vitality of mandibular central incisors. Radiograph showed radiolucency with severe alveolar bone loss localized between central incisor region of mandible, which was associated with markedly diverging roots. 

Before 1975, this lesion was probably believed to represent an atypical acanthomatous ameloblastoma or even a squamous cell carcinoma [2]. Its typical microscopic appearance justifies the name “squamous odontogenic tumor”: a stroma of mature connective tissue with islands of odontogenic epithelium. These islands have a purely squamous pattern, and the peripheral cells do not show the typical preameloblast polarization seen in ameloblastomas. Cystic degeneration in the center of the islands is a frequent finding. Prekeratin is found in some epithelial cells, and laminated calcifications may be seen inside keratin pearls [1, 2, 5–8, 11, 15–19]. The morphologic appearance of the islands of odontogenic epithelium in SOT is sometimes similar to the follicular pattern of ameloblastomas, which may lead pathologists to misclassify it as a benign odontogenic epithelial tumor, desmoplastic ameloblastoma, or ameloblastic fibroma, as occurred in some cases [4]. However, lack of polarization of peripheral cells in the epithelial islands, which is typical of ameloblastomas, is a differential criterion in favour of SOT. 

In our case, multiple islands of squamous epithelium surrounded by a mature connective tissue stroma were seen. The peripheral cells did not exhibit the polarization, characteristic of ameloblastoma, which was of major importance in establishing the diagnosis. No intraepithelial calcifications or intracellular keratinization was observed. Small microcysts were seen within the epithelial islands.

The treatment of SOT is conservative surgical removal in the form of local excision, enucleation, or thorough curettage. However, tumors located in the maxilla have to undergo a more radical treatment because of the aggressive potential of SOT in this location. Recurrence is rare and is due to incomplete removal of the tumor [4]. 

The present case adds on to the reported rare cases of SOTs with unusual localization.

## 4. Conclusion

Although three decades have passed since the establishment of the lesion as a separate entity, much remains to be learned about the SOT. A probable reason could be the comparative few reports and the common mistaken diagnosis of an acanthomatous ameloblastoma [20]. Our little experience with this type of lesion and the limited number of cases published to date, as well as the features of the case reported here and the analysis of the literature, led us to the conclusion that an individual analysis of each case is important.
